# HOW DOES THE COVID-19 PANDEMIC AFFECT LATE WORKING LIFE? EUROPEAN EMPLOYMENT TRAJECTORIES FROM 2011 TO 2021

**DOI:** 10.1093/geroni/igac059.2981

**Published:** 2022-12-20

**Authors:** Wiebke Schmitz

**Affiliations:** Unversity of Vechta, Vechta, Niedersachsen, Germany

## Abstract

**Background:**

One of the immediate impacts of the pandemic was the increase in unemployment and the reduction of working hours - especially among women, those with lower job security and in countries with a weaker social welfare system. The aging society is at further risk, which is already challenged by a growing shortage of workers and the rising costs of pensions. To understand the long-term consequences of COVID-19 for the aging workforce, this paper advances existing research by exploring from a life-course perspective how employment after the COVID-19 outbreak is anchored in larger employment histories (2011–2021).

**Methods:**

Sequence-analyses are applied using retrospective life history data combined with both COVID-19 Surveys (2020–2021) from SHARE including respondents aged 50+. Using cluster-analyses, the resulting sequences are classified into six groups. By applying descriptive-analyses, I examine how these groups differ by gender, work quality, and country.

**Results:**

Older workers – especially men – with continuous full-time employment histories are less frequently affected by unemployment and fewer working hours during COVID-19. Whereas those – especially women – with disruptive employment and part-time work histories are more frequently affected by unemployment after the outbreak of COVID-19. Respondents in countries characterized by social democratic welfare compared to liberal welfare regimes are less likely affected by unemployment during the pandemic. Discussion: The pandemic particularly puts older workers with disruptive employment histories at additional risk of labor market exit. Therefore policymakers need to address inequalities in earlier life to prevent long-term consequences of social inequality caused by the pandemic.

